# Supplementary material for: Identification of an Oxidosqualene Cyclase Gene Involved in Steroidal Triterpenoid Biosynthesis in Cordyceps farinosa
Source: Genes (Basel). 2021 May 31;12(6):848. doi: 10.3390/genes12060848 (PMC8227516; doi:10.3390/genes12060848)
Supplement: Supplementary file 1 [file genes-12-00848-s001.zip › genes-1214090-supplementary.pdf]

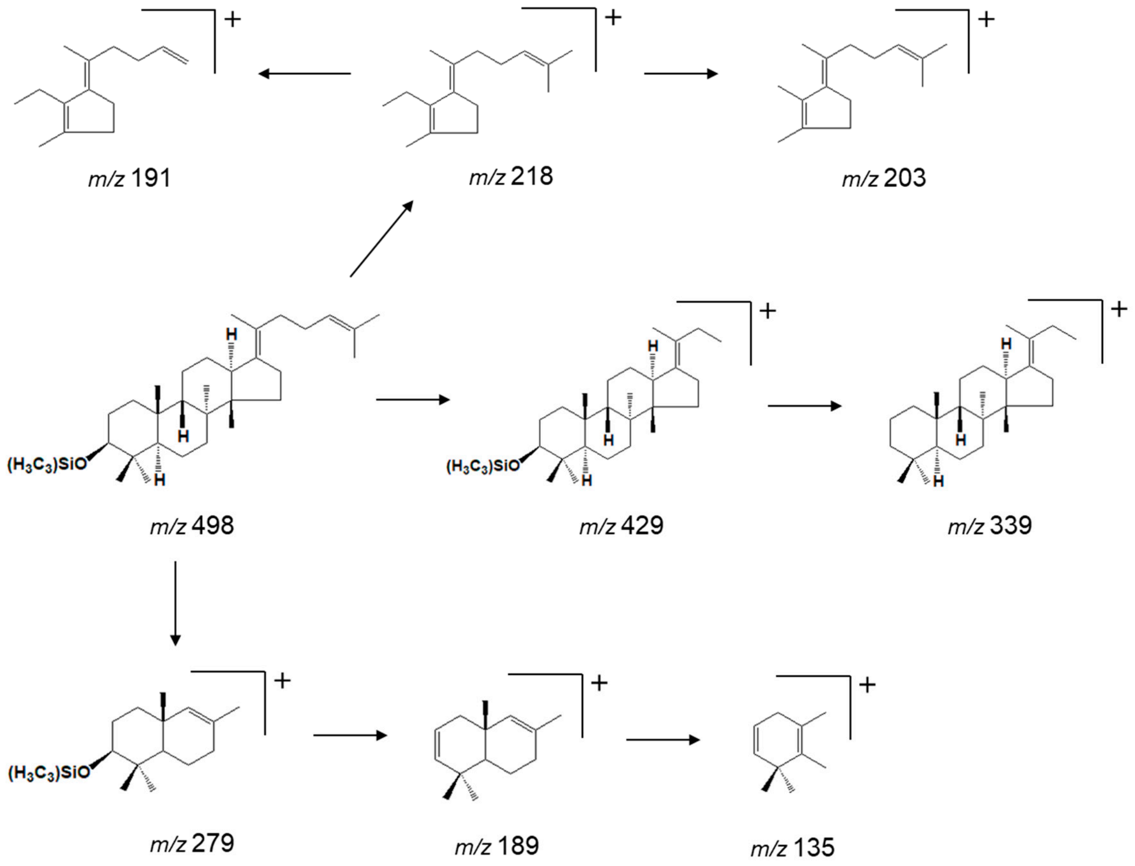

Supplemental Figure S1. Putative fragmentation mechanism of protostadienol leading to the characteristic fragment ions observed in the GC-MS spectrum.

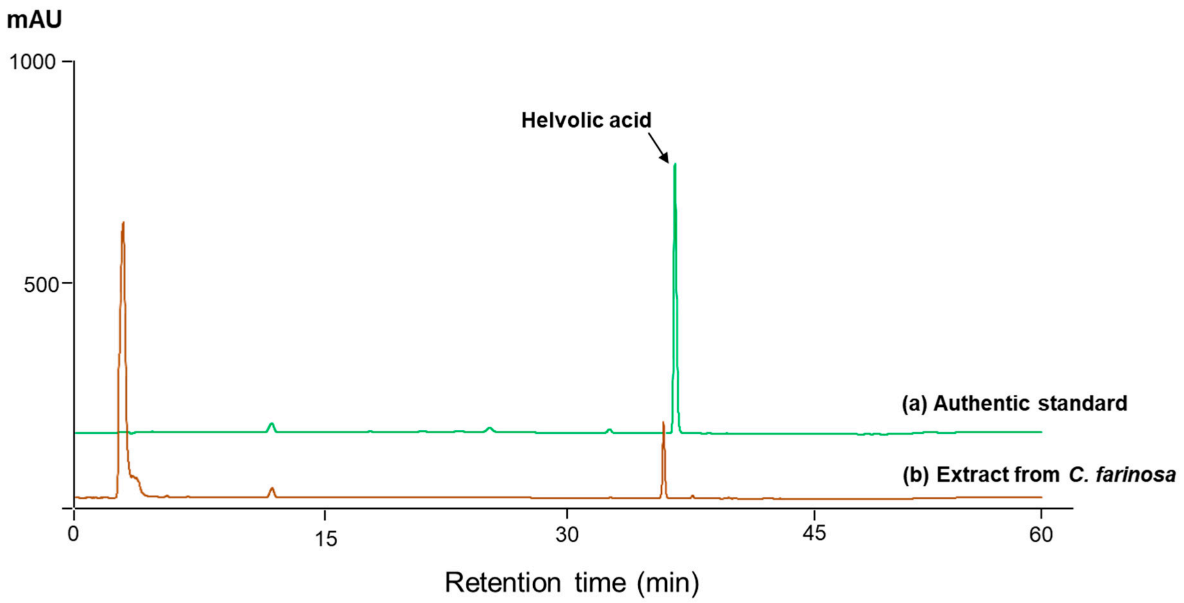

Supplemental Figure S2. HPLC analysis of helvolic acid. Two chromatograms of HPLC analysis, (a) an authentic helvolic acid and (b) the extract from *C. farinosa* mycelium.
